# Cytological Features of Kikuchi-Fujimoto Disease: A Multicenter Study of 30 Cases

**DOI:** 10.7759/cureus.83462

**Published:** 2025-05-04

**Authors:** Mamta Dwivedi, Shruti Singh, Anjani K Tripathi, Mrityunjaya Singh, Deepa Rani

**Affiliations:** 1 Pathology, King George's Medical University, Lucknow, IND; 2 General Surgery, Prasad Institute of Medical Science and Hospital, Lucknow, IND; 3 Pulmonary and Critical Care Medicine, King George's Medical University, Lucknow, IND; 4 Pathology, Sarojini Naidu Medical College, Agra, IND

**Keywords:** fine needle aspiration, histiocytic necrotizing lymphadenitis, kfd with diffuse lymphadenopathy, kikuchi-fujimoto disease, unilateral cervical lymphadenopathy

## Abstract

Background: Kikuchi-Fujimoto disease (KFD) is a frequently febrile, self-limited, subacute necrotizing lymphadenitis. It occurs predominantly in young females and is more common in Asia.

Aim: This study aims to characterize the cytomorphological spectrum of KFD through fine-needle aspiration (FNA) findings across multiple centers, highlight under-recognized diagnostic features, and refine cytologic differentials to improve diagnostic accuracy.

Materials and methods: The study group comprised a multicenter study experience of 30 cases diagnosed as KFD on cytology or subsequent histology.

Results: Cytomorphological features of 30 cases diagnosed as KFD on FNA smears were studied. Out of these 30 cases, spontaneous resolution occurred on 4 to 16 weeks of follow-up in 22 cases; in the remaining eight cases, cytologic diagnosis was confirmed on histology, supplemented by immunohistochemistry.

Conclusion: The cytological features of KFD pose diagnostic challenges, on account of its broad cytologic spectrum, though crescentic histiocytes are a constant feature. These cytological features must be interpreted in the appropriate clinical context.

## Introduction

Kikuchi-Fujimoto disease (KFD), also known as histiocytic necrotizing lymphadenitis, is a rare, self-limiting condition first described independently by Kikuchi and Fujimoto in 1972 [1,2]. It predominantly affects young adults, particularly females, and is characterized by localized lymphadenopathy, fever, and systemic symptoms that may mimic infectious or autoimmune conditions such as systemic lupus erythematosus (SLE) [3,4]. Although its etiology remains unclear, viral and autoimmune mechanisms have been postulated [5].

Cytological examination through fine-needle aspiration (FNA) has emerged as a crucial diagnostic tool for KFD, offering a minimally invasive approach to differentiate it from other causes of lymphadenopathy, including tuberculosis, lymphoma, and metastatic malignancies [6-8]. FNA cytology of KFD typically reveals a characteristic triad of necrosis, karyorrhectic debris, and a predominance of histiocytes, often with crescentic nuclei, in the absence of neutrophils [9-11]. However, due to overlapping cytological features with other necrotizing lymphadenopathies, histopathological correlation remains essential for a definitive diagnosis [12-14].

KFD continues to pose a diagnostic challenge due to its varied presentations and cytological similarities with other pathologies, such as SLE and tuberculosis. Accurate diagnosis based on FNA alone remains difficult because of the considerable cytological overlap. This study aims to systematically analyze the cytological features of KFD, correlating them with histopathology and clinical features, to enhance diagnostic accuracy and improve differentiation from other necrotizing lymphadenopathies. By identifying distinguishing features, we seek to improve the early and accurate diagnosis of this enigmatic disease.

## Materials and methods

Study design and patient selection

This was a retrospective, multicenter study conducted across three hospitals, evaluating the cytological and histopathological characteristics of KFD. A total of 30 patients diagnosed with KFD between 2020 and 2024 were included in the study. The diagnosis of KFD was confirmed based on clinical, cytological, and histopathological findings, with patients meeting the diagnostic criteria for KFD. Inclusion criteria consisted of patients who presented with cervical lymphadenopathy and were diagnosed via fine-needle aspiration cytology (FNAC) and confirmed with histopathological examination. Patients with a history of malignancy or other known causes of lymphadenopathy were excluded from the study.

Clinical data collection

Patient demographic information, including age, gender, clinical presentation, and duration of symptoms, was collected from medical records. Information on associated systemic symptoms such as fever, malaise, and night sweats was also documented. Laboratory findings, including blood counts, liver and kidney function tests, and autoimmune markers, were reviewed for correlation with the cytological and histopathological results.

FNAC and cytological evaluation

FNAC was performed using a 22- to 25-gauge needle attached to a 10 mL syringe. Smears were prepared by direct spreading and fixed in 95% ethanol for Papanicolaou (Pap) and hematoxylin and eosin (H&E) staining. Air-dried smears were stained with Giemsa stain. Ziehl-Neelsen (ZN) staining and periodic acid-Schiff (PAS) staining were performed in selected cases to rule out infectious causes of lymphadenopathy. The cytological features were assessed by two independent cytopathologists, blinded to the clinical diagnosis. In cases of diagnostic discrepancy, a consensus diagnosis was achieved after joint review. The following features were specifically evaluated: Cellular composition (e.g., histiocytes, lymphoid cells, macrophages); presence of crescentic histiocytes; evidence of necrosis (focal or diffuse); presence of apoptotic bodies and karyorrhectic debris; absence or presence of neutrophils and eosinophils; background characteristics (e.g., necrotic or reactive).

Histopathological examination

Biopsy specimens from eight patients who underwent lymph node (LN) excision were available for histopathological evaluation. The tissue samples were fixed in formalin and processed for paraffin embedding. Sections were stained with H&E, and immunohistochemistry (CD3, CD20, CD68, Ki67) was performed for further characterization of cellular components.

## Results

**Figure 1:**
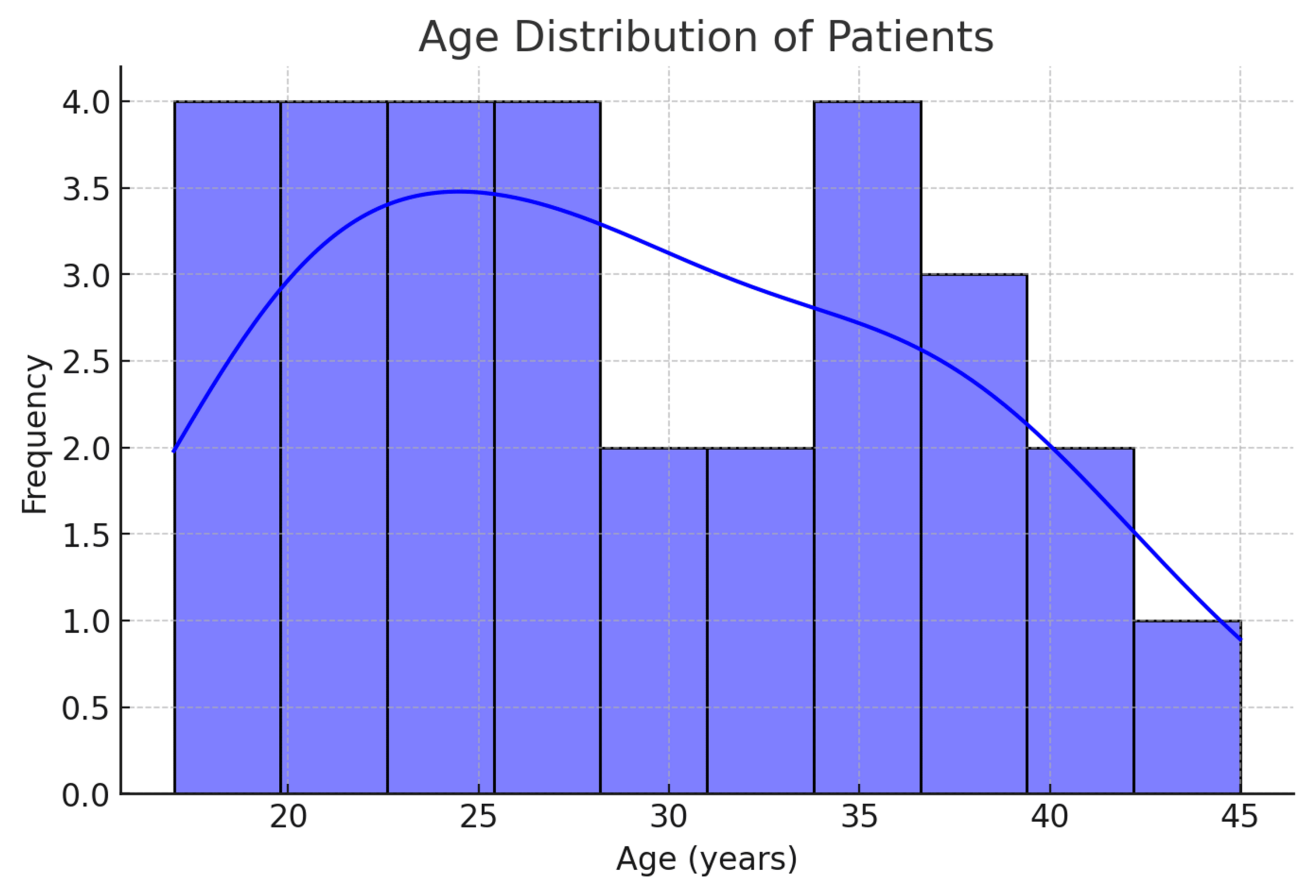
Age distribution of patients

**Figure 2:**
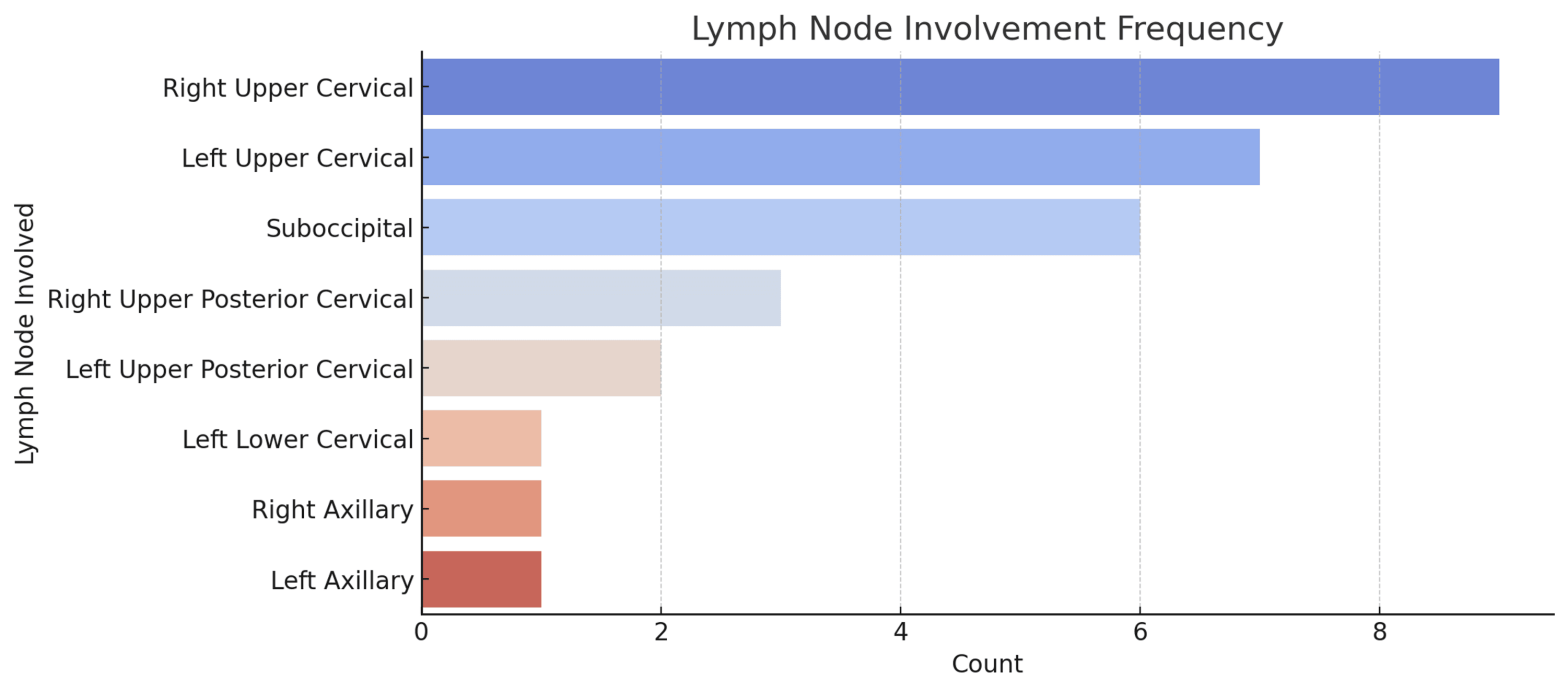
Frequency of lymph node involvement

**Figure 3:**
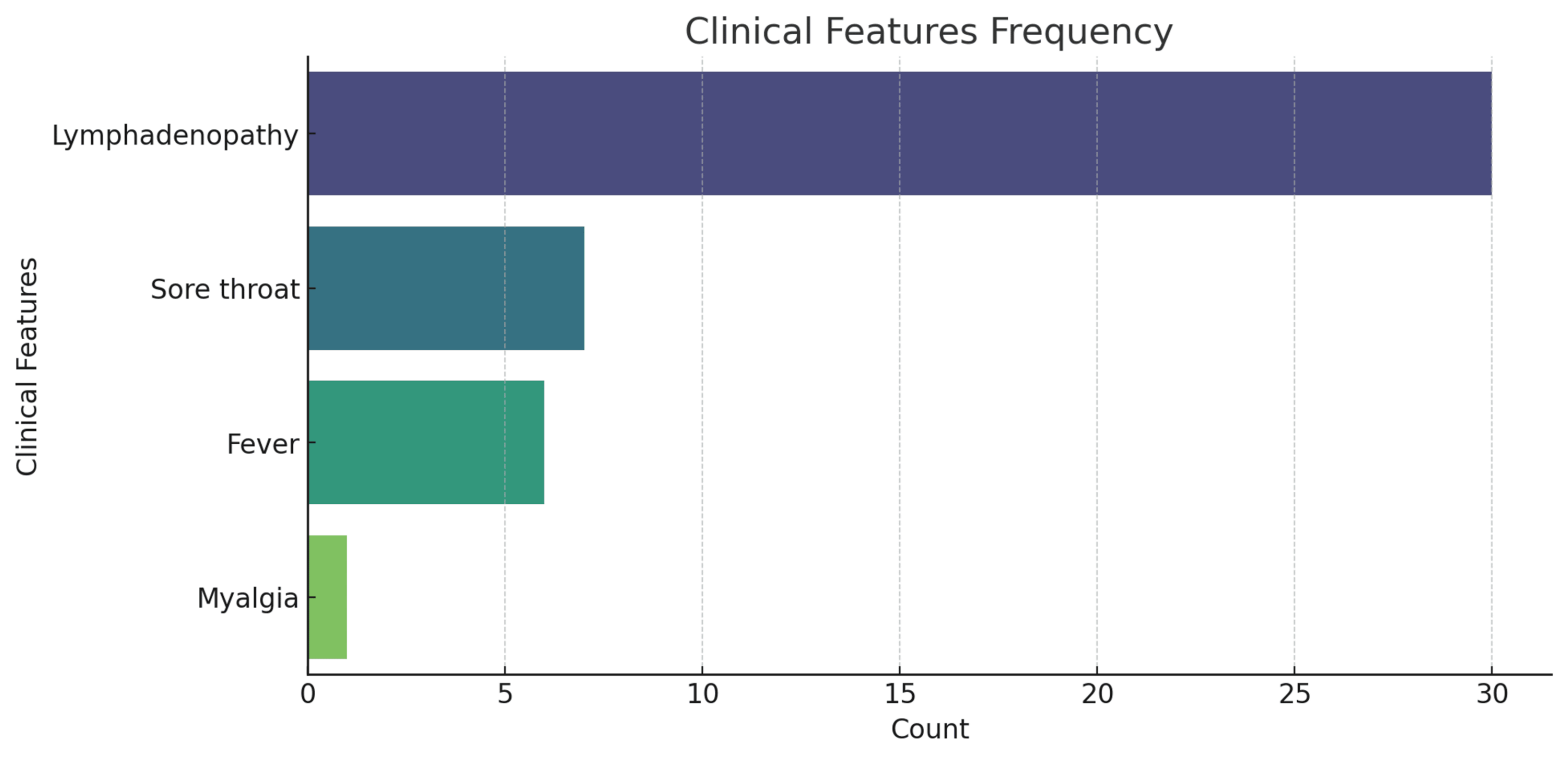
Frequency of clinical features

A total of 30 cases of lymph node (LN) enlargement were analyzed. The age of the patients ranged from 17 to 45 years (mean age: 28.6 years) (Figure 1), with a predominance of females (83.3%, n = 25) compared to males (16.7%, n = 5). The most frequently involved LN region was the right upper cervical LN (30%, n = 9), followed by the left upper cervical LN (26.7%, n = 8), and the suboccipital LN (16.7%, n = 5) (Figure 2). Other regions included the left upper posterior cervical (13.3%, n = 4), axillary (6.7%, n = 2), and multiple cervical LNs (3.3%, n = 1). The size of the LNs ranged from 0.5 cm to 2.5 cm, with a majority of cases (63.3%, n = 19) having LN enlargement between 1.0 cm and 2.0 cm. The largest LNs (2.5 × 2.0 cm) were observed in two cases - one in the left upper posterior cervical region and another in the suboccipital region. All patients (100%) presented with LN swelling as the primary symptom (Figure 3). A total of 36.7% (n = 11) had a history of mild fever and sore throat, with symptom duration ranging from 10 to 21 days. One patient (3.3%) reported myalgia as an additional symptom. The duration of LN swelling ranged from 10 to 28 days, with an average duration of 15.9 days. Females (n = 25) most commonly presented with right upper cervical LN (28%, n = 7) and left upper cervical LN (28%, n = 7). Males (n = 5) more commonly had involvement of suboccipital (40%, n = 2) and right axillary LNs (20%, n = 1) compared to females (Table 1).

**Table 1:** Demographic and clinical features of patients with Kikuchi-Fujimoto disease

S. No.	Age (Years)	Sex	Lymph Node Involved	Size (cm)	Clinical Features
1	22	F	Right Upper Cervical Lymph Node	1.5x1.0	Lymphadenopathy, Fever, Sore Throat
2	34	F	Right Upper Cervical Lymph Node	2.0x2.0	Lymphadenopathy
3	27	F	Suboccipital Lymph Node	1.0x1.0	Lymphadenopathy
4	17	F	Left Upper Cervical Lymph Node	2.0x1.5	Lymphadenopathy
5	45	F	Left Upper Posterior Cervical Lymph Node	2.5x2.0	Lymphadenopathy, Fever, Sore Throat
6	36	F	Left Lower Cervical Lymph Node	1.5x1.0	Lymphadenopathy
7	28	M	Left Upper Cervical Lymph Node	1.0x1.0	Lymphadenopathy
8	25	F	Left Upper Cervical Lymph Node	1.5x1.5	Lymphadenopathy, Fever, Sore Throat
9	27	M	Right Axillary Lymph Node	1.0x1.0	Lymphadenopathy
10	35	F	Suboccipital Lymph Node	2.0x1.5	Lymphadenopathy
11	37	F	Right Upper Cervical Lymph Node	0.5x0.5	Lymphadenopathy
12	19	F	Left Upper Cervical Lymph Node	2.0x1.5	Lymphadenopathy, Fever, Sore Throat
13	30	F	Right Upper Posterior Cervical Lymph Node	1.5x1.0	Lymphadenopathy
14	39	F	Right Upper Cervical Lymph Node	1.0x1.0	Lymphadenopathy
15	28	F	Left Axillary Lymph Node	1.0x1.0	Lymphadenopathy
16	22	F	Left Upper Cervical Lymph Node	1.0x1.0	Lymphadenopathy
17	25	F	Suboccipital Lymph Node	2.0x1.0	Lymphadenopathy, Sore Throat
18	20	F	Right Upper Cervical Lymph Node	1.5x1.0	Lymphadenopathy
19	40	F	Left Upper Cervical Lymph Node	1.5x1.5	Lymphadenopathy
20	23	F	Right Upper Cervical Lymph Node	2.0x1.0	Lymphadenopathy
21	31	M	Suboccipital Lymph Node	0.5x0.5	Lymphadenopathy, Fever
22	23	F	Right Upper Posterior Cervical Lymph Node	1.5x1.5	Lymphadenopathy, Sore Throat
23	18	F	Suboccipital Lymph Node	1.5x1.0	Lymphadenopathy
24	32	M	Right Upper Cervical Lymph Node	0.5x0.5	Lymphadenopathy
25	29	F	Left Upper Posterior Cervical Lymph Node	2.0x1.0	Lymphadenopathy
26	35	F	Right Upper Cervical Lymph Node	1.5x1.5	Lymphadenopathy, Myalgia
27	21	F	Right Upper Posterior Cervical Lymph Node	1.5x1.0	Lymphadenopathy, Fever
28	39	F	Right Upper Cervical Lymph Node	2.0x2.0	Lymphadenopathy, Sore Throat
29	19	F	Suboccipital Lymph Node	2.5x2.0	Lymphadenopathy
30	42	F	Left Upper Cervical Lymph Node	0.5x0.5	Lymphadenopathy

**Figure 4:**
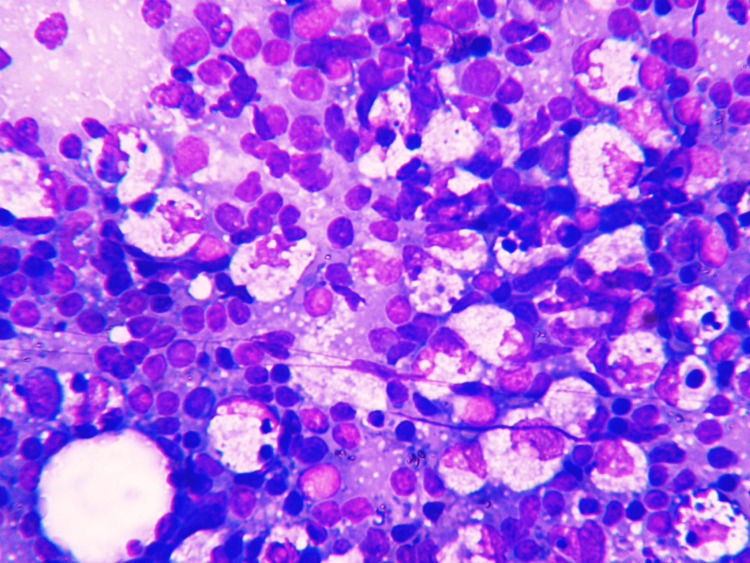
Highly cellular smear shows crescentic histiocytes admixed with lymphoid cells, along with apoptotic bodies and karyorrhectic debris over a necrotic background (40x, May-Grünwald-Giemsa stain)

**Figure 5:**
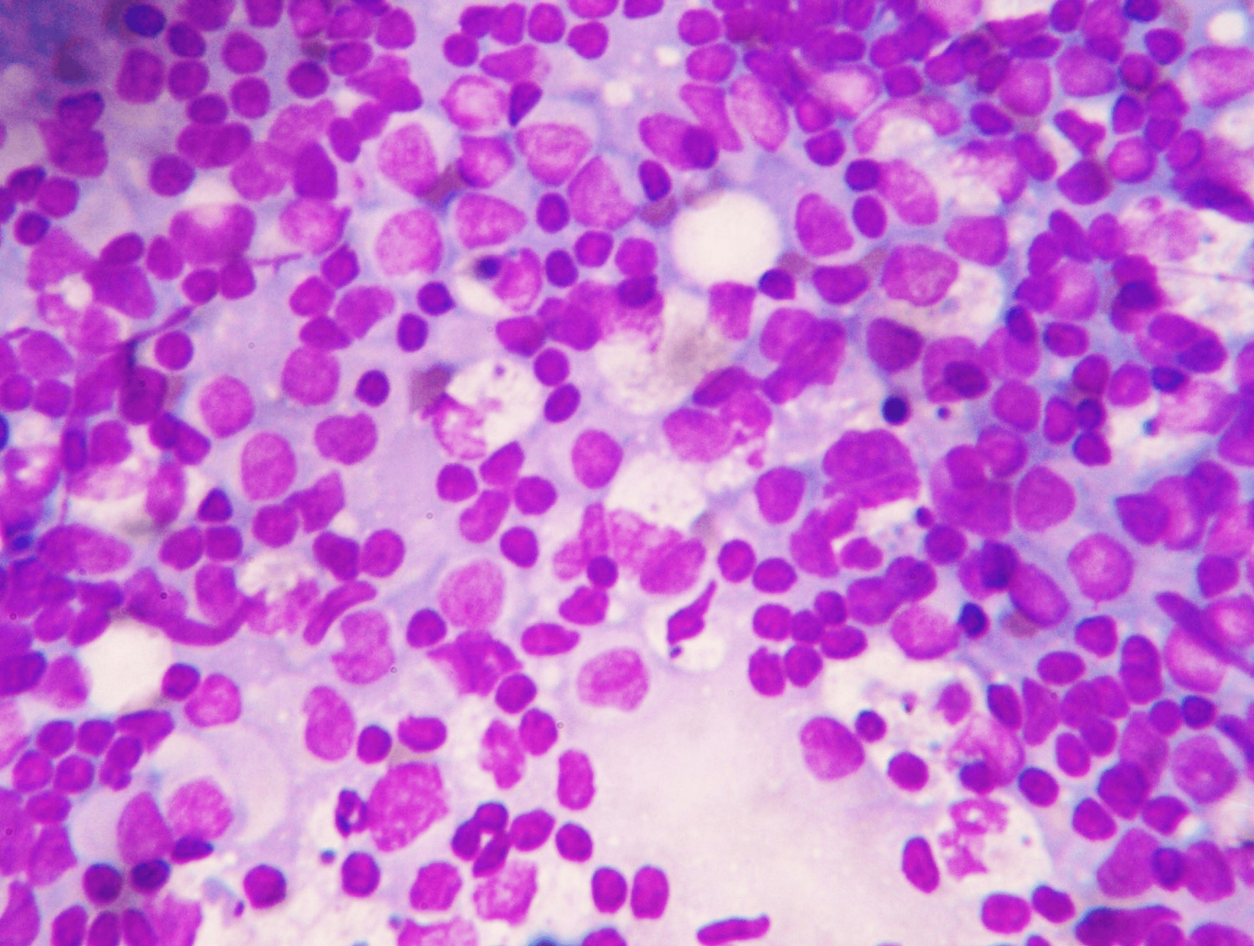
Smear shows high cellularity with crescentic histiocytes admixed with lymphoid cells, along with apoptotic bodies (40x, May-Grünwald-Giemsa stain)

All 30 patients were subjected to FNAC. The cytological features and diagnosis are summarized in Table 2. Smears revealed predominantly lymphoid tissue. Cellularity was high in 23 cases (76.7%), while seven cases (23.3%) presented with moderate cellularity. Focal areas of necrosis were seen in 26 cases (86.7%). Numerous histiocytes were seen admixed with lymphoid cells. Crescentic histiocytes were a consistent finding in all cases (100%), although their numbers varied from a few to many. Apoptotic bodies and karyorrhectic debris were absent in two cases (6.7%), while in the remaining 28 cases (93.3%), their number varied from scanty to abundant (Figures 4-5). Neutrophils and eosinophils were inconspicuous in 24 cases (80%), while they were present in six cases (20%). Occasional plasma cells were seen in three cases (10%).

**Table 2:** Cytological profile and diagnostic categorization of Kikuchi-Fujimoto disease (KFD)

S. No.	Cellularity	Focal Area of Necrosis	Crescentic Histiocytes	Apoptotic Bodies	Karyorrhectic Debris	Neutrophils and Eosinophils	Cytological Diagnosis
1	High	Present	Many	Abundant	Abundant	Absent	Consistent with KFD
2	High	Present	Moderate	Very Few	Very Few	Absent	Consistent with KFD
3	Moderate	Present	Few	Few	Few	Very Few	Suggestive of KFD
4	High	Present	Many	Abundant	Abundant	Absent	Highly Suggestive of KFD
5	High	Absent	Many	Very Few	Very Few	Absent	Consistent with KFD
6	High	Present	Many	Very Few	Very Few	Absent	Consistent with KFD
7.	High	Present	Few	Absent	Absent	Very Few	Suggestive of KFD
8.	High	Absent	Many	Very Few	Very Few	Absent	Suggestive of KFD
9.	High	Present	Many	Abundant	Abundant	Absent	Consistent with KFD
10.	High	Present	Moderate	Moderate	Moderate	Absent	Consistent with KFD
11.	Moderate	Present	Moderate	Moderate	Moderate	Absent	Consistent with KFD
12.	Moderate	Present	Moderate	Moderate	Moderate	Absent	Consistent with KFD
13.	High	Present	Many	Abundant	Abundant	Absent	Consistent with KFD
14.	High	Absent	Many	Abundant	Abundant	Absent	Consistent with KFD
15.	High	Present	Many	Absent	Abundant	Very Few	Suggestive of KFD
16.	High	Present	Many	Moderate	Abundant	Absent	Consistent with KFD
17.	High	Present	Many	Abundant	Moderate	Absent	Consistent with KFD
18.	High	Absent	few	Few	Absent	Absent	Highly Suggestive of KFD
19.	Moderate	Present	Many	Moderate	Moderate	Absent	Highly Suggestive of KFD
20.	High	Present	Many	Abundant	Few	Absent	Consistent with KFD
21.	High	Present	Moderate	Very Few	Moderate	Absent	Highly Suggestive of KFD
22.	High	Present	Many	Abundant	Few	Absent	Highly Suggestive of KFD
23.	High	Present	Many	Abundant	Very Few	Absent	Consistent with KFD
24.	Moderate	Present	Few	Few	Moderate	Very Few	Suggestive of KFD
25.	High	Present	few	Abundant	Abundant	Absent	Consistent with KFD
26.	Moderate	Present	Moderate	Very Few	Moderate	Very Few	Suggestive of KFD
27.	High	Present	Many	Moderate	Few	Absent	Consistent with KFD
28.	Moderate	Present	Many	Few	Few	Very Few	Suggestive of KFD
29.	High	Present	few	Abundant	Moderate	Absent	Highly Suggestive of KFD
30.	High	Present	Moderate	Abundant	Abundant	Absent	Highly Suggestive of KFD

**Figure 6:**
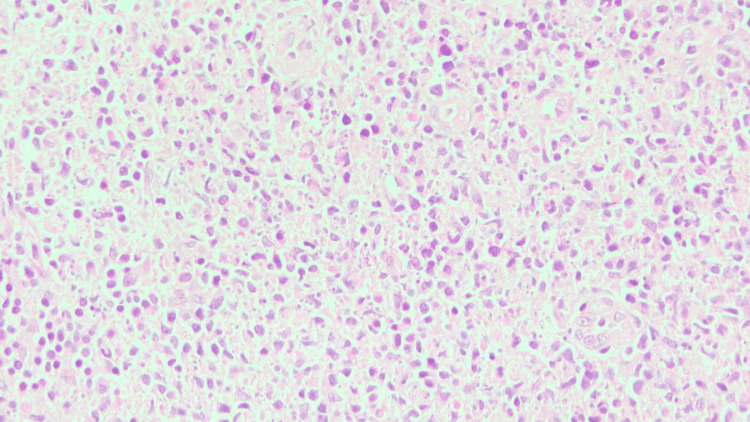
Crescentic histiocytes are seen admixed with lymphoid cells, along with apoptotic bodies and karyorrhectic debris over a necrotic background (Histology - 40x, hematoxylin and eosin stain)

**Figure 7:**
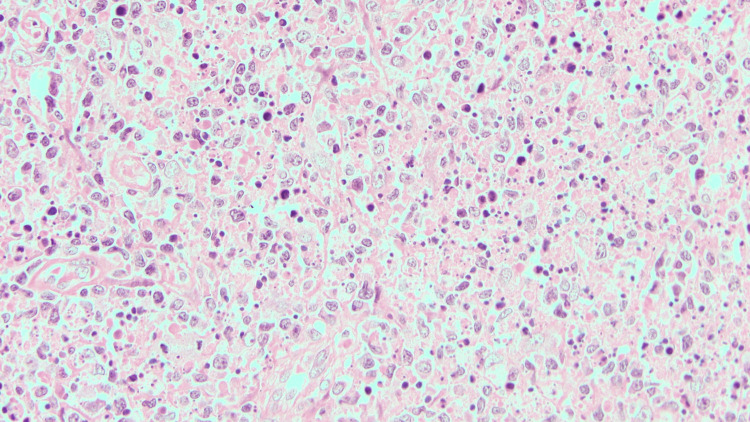
Crescentic histiocytes are seen admixed with lymphoid cells, along with apoptotic bodies and karyorrhectic debris over a necrotic background (Histology - 40x, hematoxylin and eosin stain)

Lymphadenopathy regressed in 22 cases (73.3%) during follow-up, while the remaining eight cases (26.7%) were subjected to LN biopsy. Histology revealed focal paracortical necrosis with abundant karyorrhectic nuclear debris. Few apoptotic bodies and crescentic histiocytes were noted at the edge of the necrotic area. The periphery of the necrotic areas also revealed nests of plasmacytoid monocytes and immunoblasts (Figures 6-7).

**Figure 8:**
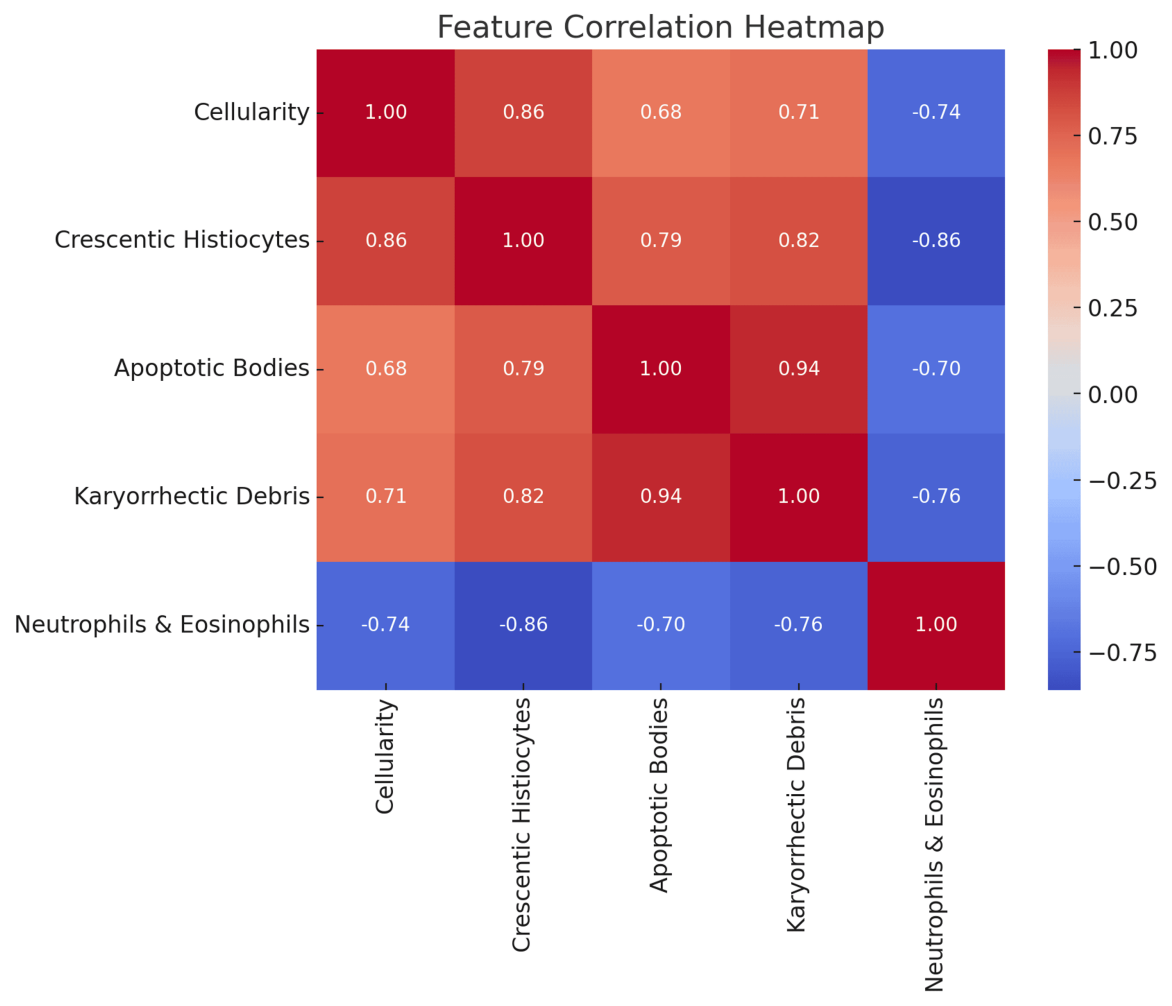
Heatmap highlighting the correlations between different cytological features

The heatmap highlights the interdependencies between various histopathological features found in KFD. Crescentic histiocytes, apoptotic bodies, and karyorrhectic debris are positively correlated, suggesting they may be key diagnostic markers occurring together. Neutrophils and eosinophils show negative correlations with most other features, implying their presence is inversely related to features like crescentic histiocytes and apoptotic bodies (Figure 8).

**Figure 9:**
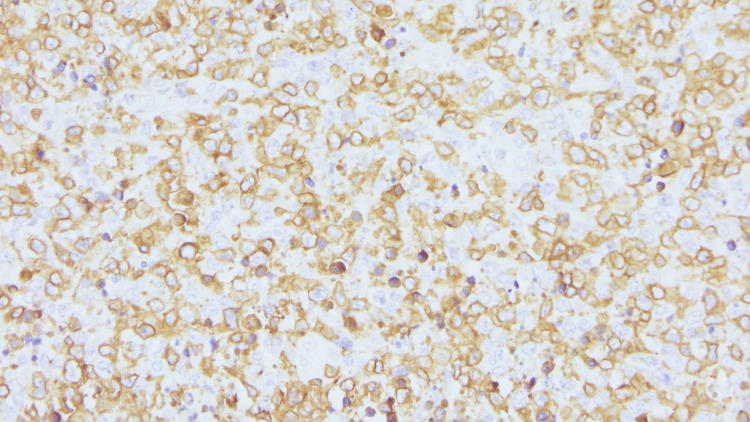
Immunohistochemistry (40x magnification) showing CD3 positivity in reactive T lymphocytes

**Figure 10:**
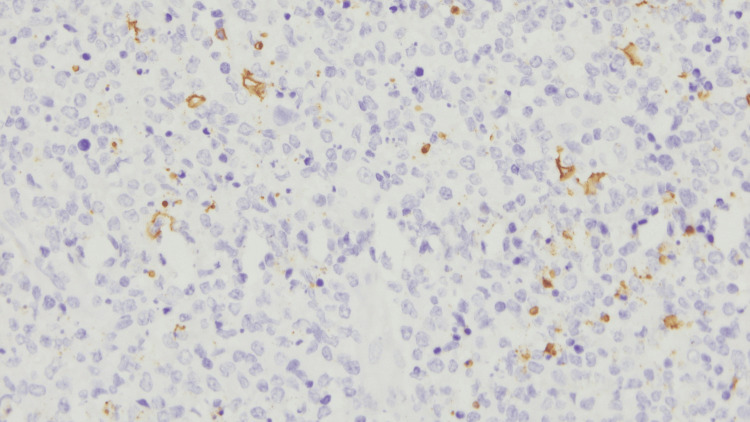
CD20 positivity observed in B lymphocytes (Immunohistochemistry - 40x)

**Figure 11:**
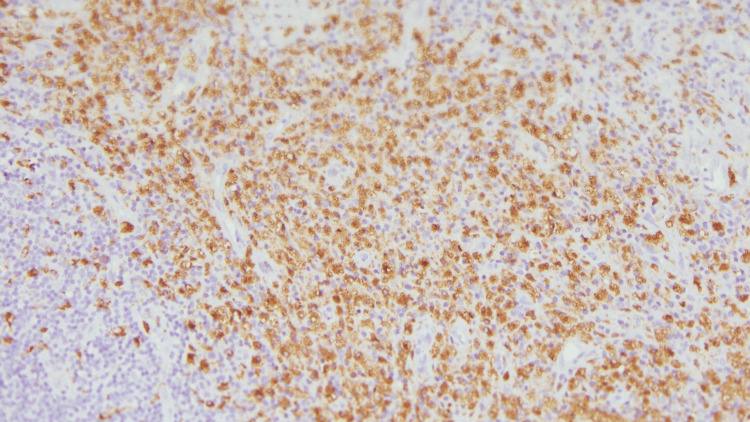
CD68 positivity observed in histiocytes (Immunohistochemistry - 40x)

**Figure 12:**
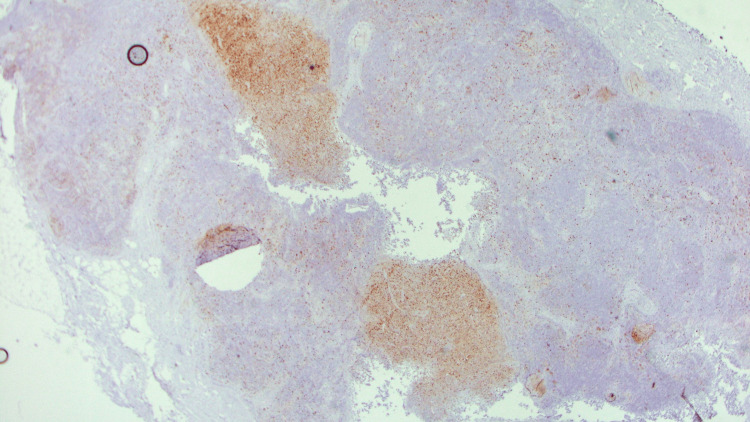
Ki67 positivity observed in germinal centers (Immunohistochemistry - 10x)

Immunohistochemistry (IHC) was performed in all eight cases (100% of biopsy cases, 26.7% of total cases). IHC revealed CD3 positivity in reactive T lymphocytes, CD20 positivity in B lymphocytes, and CD68 positivity in histiocytes. Ki67 positivity was high in the germinal centers (Figures 9-12).

## Discussion

KFD was first described in 1972 by Kikuchi and Fujimoto independently as a case of lymphadenitis with extensive nuclear debris and histiocytic proliferation [1,2]. The etiology of this disease is not known. However, various etiological agents like human herpes virus (HHV6 and HHV8), herpes simplex virus, adenovirus, parvovirus B19, cytomegalovirus, varicella zoster, dengue virus, bacteria such as *Mycobacterium azulgai*, *Yersinia*, and protozoa have been linked to the disease [15,16]. Disease has been reported worldwide, with a higher prevalence among Asians. The important clinical features of KFD are female predominance with a mean age of 30 years and painless lymphadenopathy mostly in the cervical region [14]. Other reported features are fever, myalgia, sore throat, localized pain, and mild leucopenia [17,18]. In our study, the male-to-female ratio was 1:6.5. The patients' ages ranged from 17 to 45 years. Among them, 22 cases had cervical lymphadenopathy, six presented with suboccipital LN enlargement, and two cases had axillary lymphadenopathy.

Yoo et al. reported 30 cases of KFD. In their series, all the cases revealed hypercellularity with abundant extracellular karyorrhectic debris [9]. Han et al. revealed low cellularity in five cases out of 10 and high cellularity in the remaining five cases. Karyorrhectic debris was seen in three out of 10 cases [11]. In our study, high cellularity was observed in 23 cases, while seven cases exhibited moderate cellularity. Karyorrhectic debris was present in 28 cases but absent in only two cases. Karyorrhectic debris is one of the characteristic cytological features of KFD [10,19]. It appears as irregularly shaped nuclear fragments without cytoplasm. Apoptotic bodies are another characteristic feature of KFD, appearing as dense eosinophilic bodies. In our study, they were observed in 28 out of 30 cases. In the study by Han et al., apoptotic bodies were present in all 10 cases [11].

Crescentic histiocytes are recognized as one of the most characteristic cells in KFD [10]. Han et al. reported crescentic histiocytes in 80% of their case series [11]. In our study, crescentic histiocytes were a consistent finding in all cases; however, their numbers varied from a few to many. These histiocytes have eccentrically placed crescent-shaped nuclei and pale cytoplasm containing ingested nuclear debris [13]. They must be differentiated from the tingible body macrophages, which have centrally placed, round to ovoid nuclei with ingested debris. Therefore, the presence of crescentic histiocytes in cytology serves as an important diagnostic indicator. Neutrophils and eosinophils were inconspicuous in 24 cases but were observed in six cases, likely as blood-derived elements.

In our study, focal areas of necrosis with a granular background were observed in 26 out of 30 cases. This type of necrosis must be distinguished from caseous necrosis, which is characteristic of tubercular lesions. Han et al. and Tsang and Chan also reported no cases of caseous necrotic background in KFD in their studies [11,12]. The main differential diagnoses of KFD on cytology include SLE, reactive follicular hyperplasia, infectious diseases such as tuberculosis, LN infarction, and malignant lymphoma [8,20,21].

SLE is particularly challenging to distinguish from KFD, as both conditions exhibit necrosis, nuclear debris, apoptotic bodies, lipid-laden histiocytes, and a lack of neutrophils and eosinophils. However, hematoxylin bodies, plasma cells, clinical examination, and laboratory evaluation for antinuclear antibodies are helpful in differentiating SLE from KFD [7,22]. Imamura et al. also noted histopathological similarities between KFD and SLE [23].

Distinguishing KFD from reactive follicular hyperplasia can be difficult due to the limitations of cytology. However, the presence of apoptotic bodies and crescentic histiocytes serves as a helpful diagnostic clue. In contrast, necrotizing lymphadenitis associated with tuberculosis is characterized by epithelioid histiocytes, granuloma formation, and occasional Langhans-type giant cells, none of which are present in KFD.

LN infarction is marked by ischemic necrosis but lacks nuclear debris. Additionally, the proliferation of immunoblasts and plasmacytoid dendritic cells during the proliferative phase of KFD may closely resemble non-Hodgkin lymphoma [24]. Ancillary studies are essential for distinguishing such cases.

Histological examination of the eight cases that underwent LN biopsy revealed further evidence supporting the diagnosis of KFD, with focal paracortical necrosis and abundant karyorrhectic nuclear debris. These histological findings are typical of KFD and are key to distinguishing it from other causes of lymphadenitis [25]. Immunohistochemical analysis of all eight KFD cases revealed a significant presence of T cells and macrophages within the lesions. CD3 expression was predominantly observed in paracortical lymphoid cells, whereas CD20-positive B cells were sparsely distributed. The majority of infiltrating cells were histiocytes, which exhibited positive staining for CD68. The high Ki67 positivity in the germinal centers reflects an active proliferative process, consistent with the heightened immune activity seen in KFD [26,27].

This study has several limitations. While the multicenter design enhances the generalizability of our findings, the sample size remains modest due to the rarity of KFD. The absence of long-term clinical follow-up data restricts our ability to assess disease recurrence or progression. Additionally, although two pathologists independently reviewed the FNAC smears and resolved discrepancies through consensus, we did not conduct formal interobserver variability analysis, such as using kappa statistics. Selection bias could also be a factor, as cases with atypical or inconclusive FNAC findings may have been underrepresented. Future studies with larger cohorts, formal reproducibility analysis, and clinical follow-up are needed to validate and expand on these findings.

## Conclusions

KFD can be diagnosed cytologically, and the presence of crescentic histiocytes, karyorrhectic debris, and apoptotic bodies is a critical diagnostic feature. Although FNAC is valuable, it has limitations, particularly in atypical or subtle cases, and should be complemented by histopathology and immunohistochemistry when necessary. Awareness of the cytological spectrum of KFD is crucial for accurate diagnosis, especially in less classical cases, to avoid unnecessary biopsies and overtreatment. A multidisciplinary approach, incorporating clinical, cytological, and histopathological findings, enhances diagnostic accuracy and ensures optimal management for patients with KFD.
